# Pseudo dual-energy CT-derived iodine mapping using single-energy CT data based on a convolution neural network

**DOI:** 10.1259/bjro.20220059

**Published:** 2023-10-18

**Authors:** Yuki Yuasa, Takehiro Shiinoki, Koya Fujimoto, Hidekazu Tanaka

**Affiliations:** 1 Department of Radiation Oncology, Graduate School of Medicine, Yamaguchi University, Ube, Yamaguchi, Japan

## Abstract

**Objective:**

The objectives of this study are: (1) to develop a convolutional neural network model that yields pseudo high-energy CT (CT_pseudo_high_) from simple image processed low-energy CT (CT_low_) images, and (2) to create a pseudo iodine map (IM_pseudo_) and pseudo virtual non-contrast (VNC_pseudo_) images for thoracic and abdominal regions.

**Methods:**

Eighty patients who underwent dual-energy CT (DECT) examinations were enrolled. The data obtained from 55, 5, and 20 patients were used for training, validation, and testing, respectively. The ResUnet model was used for image generation model and was trained using CT_low_ and high-energy CT (CT_high_) images. The proposed model performance was evaluated by calculating the CT values, image noise, mean absolute errors (MAEs), and histogram intersections (HIs).

**Results:**

The mean difference in the CT values between CT_pseudo_high_ and CT_high_ images were less than 6 Hounsfield unit (HU) for all evaluating patients. The image noise of CT_pseudo_high_ was significantly lower than that of CT_high_. The mean MAEs was less than 15 HU, and HIs were almost 1.000 for all the patients. The evaluation metrics of IM and VNC exhibited the same tendency as that of the comparison between CT_pseudo_high_ and CT_high_ images.

**Conclusions:**

Our results indicated that the proposed model enables to obtain the DECT images and material-specific images from only single-energy CT images.

**Advances in knowledges:**

We constructed the CNN-based model which can generate pseudo DECT image and DECT-derived material-specific image using only simple image-processed CT_low_ images for the thoracic and abdominal regions.

## Introduction

Dual-energy computed tomography (DECT) has gained significant attention in the field of diagnostic imaging. In addition to CT images, DECT imaging can provide material-specific images including iodine maps (IMs), electron density maps, virtual monochromatic images, and virtual unenhanced images.^1^ Those images are beneficial to diagnostic imaging, evaluation of organ perfusion, reducing of artifacts, and detection of tumour.^2,3^ In particular, an IM helps in the detection of organ perfusion changes.^4^ Several researchers have reported the applicability of IM for lung perfusion evaluations and have concluded that IM is helpful to detect pulmonary embolism.^5,6^ Additionally, many reports have promoted the use of IM to evaluate liver perfusion and assess the treatment response.^7,8^ Furthermore, the effectiveness of virtual monochromatic and unenhanced images in the clinical also has been reported.^9^

Although the DECT images are effective for various cases, a special DECT scanner is required to obtain them. However, DECT scanners are not widely installed such as single-energy CT (SECT) scanners. To acquire DECT images using a SECT scanner, multiple CT scans with different X-ray spectra are required, and they induce: [1] an excess of radiation dose, [2] a discrepancy in the contrast-phase between scans, and [3] an adverse effect of the patient’s movements during scan. Because of these problems, it is difficult to acquire high-quality DECT images without using a special DECT scanner.

Recently, deep-learning methods have been extensively applied in medical image processing. In particular, the convolutional neural network (CNN) is widely used for the image generation, segmentation, and classification.^10,11^ Several researchers reported methods to synthesize DECT using the deep-learning model. Zhao et al generated the synthetic DECT images based on the differences predicted between the CT_high_ and CT_low_ images using deep U-Net models.^12^ Although their model can generate highly accurate CT_high_ images, they require multiple deep-learning models and an indirect prediction approach based on the calculation of differential images from CT_high_ and CT_low_ images. Owing to their complexity, these methods may not be suitable for clinical applications. Therefore, a simplified image processing-based method is required. Li et al implemented several connected deep CNN models to obtain a synthetic high-energy CT (CT_high_) image from a low-energy CT (CT_low_) image of the thoracic region and evaluated the accuracy of the model by measuring the pixel value for only one patient.^13^ However, they evaluated the accuracy of the model by focusing only on the CT value, and the image quality of synthetic image was not evaluated. The evaluations of image quality are required to evaluate the accuracy of model in detail.

Furthermore, there are few reports regarding the generation of pseudo-DECT (DECT_pseudo_)-derived images such as virtual non-contrast (VNC) images and IMs using deep-learning methods. The DECT_pseudo_-derived images from only SECT images may help to improve the diagnostic ability (*e.g.,* pulmonary embolism, liver tumours).

The objectives of this study are to develop a CNN-based model that yields pseudo high-energy CT (CT_pseudo_high_) images based on the simple image processed CT_low_ images and to create an VNC image and IM using CT_pseudo_high_ and CT_low_ images for the thoracic and abdominal regions. Furthermore, image similarity and pixel value of the synthetic images were investigated to evaluate the accuracy of proposed model.

## Methods

### Data acquisition and image processing

In this study, we used the paired contrast enhanced CT_high_ and CT_low_ images to train and evaluate the proposed model. The use of the patient data was approved by Institutional Review Board of Yamaguchi University (approval number: H2019-080).

Eighty patients who underwent contrast-enhanced CT between January 2018 and February 2022 were enrolled in the thoracic and abdominal regions, respectively. We employed the data of 55, 5, and 20 patients for training, validation, and testing, respectively. The training data consisted of 1,7070 and 1,3223 image slices of the thoracic and abdominal regions, respectively. The contrast-enhanced CT_high_ and CT_low_ images were acquired on a dual-source DECT scanner (SOMATOM Force; Siemens Healthineers, Forchheim, Germany). The tube voltage was 90 and Sn 150 kV (150 kV with tin filter) for the thoracic region, 100 kV and Sn 150 kV for the abdominal region. The tube current was adjusted using CT-auto exposure control. All the CT images were reconstructed with a 512 × 512 matrix and a slice thickness of 1 mm using the iterative image reconstruction method.

For the contrast agent administration, an iodine contrast agent was injected at the rate of 3 to 5 ml s^−1^. The total volume of the contrast agent was adapted to the patient’s body weight at 2 ml/kg. The CT images were acquired using bolus tracking method, and the starting threshold value of the region of interest (ROI) was set to 100 Hounsfield Units (HU) on the pulmonary artery for the thoracic region and abdominal aorta for the abdominal regions. The data acquisition delays were 10 and 45 s after reaching the threshold values for the thoracic and abdominal regions, respectively.

Using the commercial workstation (SyngoVIA cliant; ver. 4.1, Siemens Healthineers, Forchheim, Germany), the ground truth VNC (VNC_truth_) images and ground truth IM (IM_truth_) were calculated with CT_high_ and CT_low_ images to evaluate the accuracy of the model.

### Model training and evaluation

**Figure 1.**
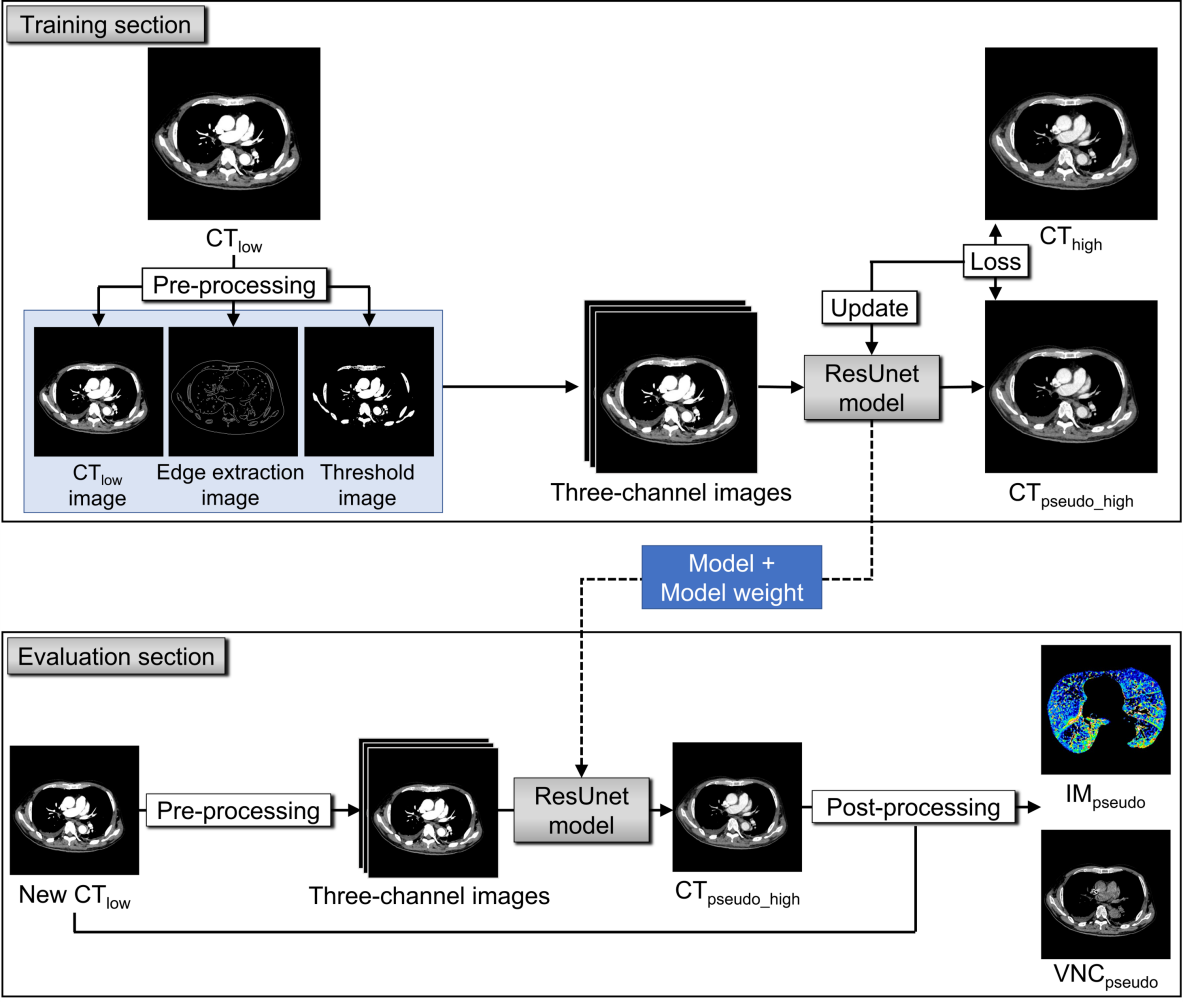
Schematic of the training and evaluation of the developed model. First, pre-image processing for CT_low_ was carried out to define the input data for the model. The three-channel images created by stacking the original CT_low_ image, extracted edges, and threshold-processed CT_low_ images, which were used as input data. Second, the ResUnet model was trained using the image data from 30 patients for the thoracic and abdominal regions. The model weights were updated while minimizing the difference between the generated CT_pseudo_high_ images and the corresponding ground-truth CT_high_ images. Finally, the trained model was evaluated using the new CT_low_ image data of 10 patients. The accuracy of the model was evaluated by comparing the obtained CT_pseudo_high_ images with the corresponding ground-truth CT_high_ images. Similarly, the VNC_pseudo_ and IM_pseudo_ images were compared with the corresponding VNC_truth_ and IM_truth_.

Figure 1 shows the workflow in this study. The ResUnet model was used as the image generation model. The architecture and implementation details of the ResUnet are provided in Supplementary Material 1. This study has the training and evaluation sections. The proposed model was trained using the paired CT_high_ and CT_low_ images in the training section. For the evaluation section, the new CT_low_ images, which were not used for the training were input into the trained model, and CT_pseudo_high_ images were output. The steps for model training and evaluation are as follows.

Supplementary S1.

In the first step, pre-image processing for CT_low_ was carried out to create the input data of the model. To prevent adverse effect from the non-anatomical regions such as the CT couch and patient immobilization devices, they were removed using the binary mask image of the patient’s body. The binary mask image was created using 3D Slicer (ver. 4.7.0; Brigham and Females’s Hospital, Boston, MA, USA). Regions outside the mask were set to a CT value of −1000 Hounsfield unit (HU). The accuracy and generalization performance of the deep learning model can be improved by data augmentation.^14^ Therefore, simple image processing-based data augmentation was applied to the original CT_low_ images to improve the accuracy and generalization performance of the model. The resulting data-augmented three-channel images were used as the input data. to improve the accuracy and versatility of the model. The resulting data-augmented three-channel images were used as the input data. For the extraction of edges, the Sobel filter was applied to extract the edge of the organ. For the threshold processing, the areas with CT values above 100 HU were extracted for the CT_low_ images. The CT value for threshold processing was determined according to the previous studies^15,16^

In the second step, the ResUnet model was trained on a graphic processing unit (GPU; NVIDIA GeForce GTX 1080Ti) using the training data. The model was separately trained for the thoracic and abdominal regions. The three-channel images were input into the model, and the CT_pseudo_high_ images were obtained as outputs. The model weights were updated while minimizing the difference between the generated CT_pseudo_high_ images and the corresponding ground-truth CT_high_ images.

In the final step, the accuracy of the model was evaluated using the new CT_low_ image data of 10 patients. The new CT_pseudo_high_ images were generated from the new CT_low_ images using the trained ResUnet model and were compared with the corresponding ground-truth CT_high_ images.

After that, the pseudo-VNC (VNC_pseudo_) images and pseudo-IM (IM_pseudo_) were calculated using the CT_pseudo_high_ and CT_low_ images using the workstation for the 10 patients of thoracic and abdominal regions. For IM_pseudo_, the lung and liver areas were segmented to only assess iodine distribution of the lungs and livers. These images were compared with corresponding IM_truth_.

### Quantitative evaluation metrics

To evaluate the accuracy of the proposed model, the mean CT value, image noise, MAE, and histogram intersection (HI) were calculated to compare CT_pseudo_high_ with the ground truth CT_high_ images.

The mean CT value in the ROI of 10 × 10 pixels was calculated using ImageJ software.^17^ For the thoracic region, ROIs were manually positioned on the pulmonary artery, pulmonary vein, ascending aorta, lung, spine, and muscle. For the abdominal region, those were positioned on the abdominal aorta, portal vein, liver, spleen, pancreas, kidney, and stomach. The difference in CT values between CT_high_ and CT_pseudo_high_ images was calculated.

The image noise was defined as the standard deviation (SD) of CT values in the ROI of 10 × 10 pixels. For the thoracic region, the ROI was positioned on the uniform region of the ascending aorta. For the abdominal region, it was positioned on the uniform region of the liver parenchyma.^18,19^ The difference in image noise between CT_high_ and CT_pseudo_high_ images was calculated.

The MAE between CT_high_ and CT_pseudo_high_ was calculated for the thoracic and abdominal regions. Furthermore, to evaluate the similarities between images, the HI was calculated as follows:

(1) $$HI=\;\frac{\sum_{j=1}^{n}min\left(H1_{j},\;H2_{j}\right)}{\sum_{j=1}^{n}H1_{j}}$$

where H1_j_ and H2_j_ represent the histogram values of CT_high_ and CT_pseudo_high_ for bin number j, respectively.^20^ The bin size of the histogram is 5 HU.

Similarly, the VMC_pseudo_ was compared with the VNC_truth_ using the CT value, image noise, MAE, and HI. For the comparison of IM, because IM was segmented to evaluate only the iodine distribution of lung and liver, IM_pseudo_ was compared with the IM_truth_ using only the MAE and HI.

To assess the difference in CT value and image noise, the statistical analysis was performed with the Shapiro-Wilk and Wilcoxon rank sum tests. All *p*-values less than 0.05 were considered to be statistically significant.

## Results

### Comparison between ground truth and pseudo-images

**Figure 2.**
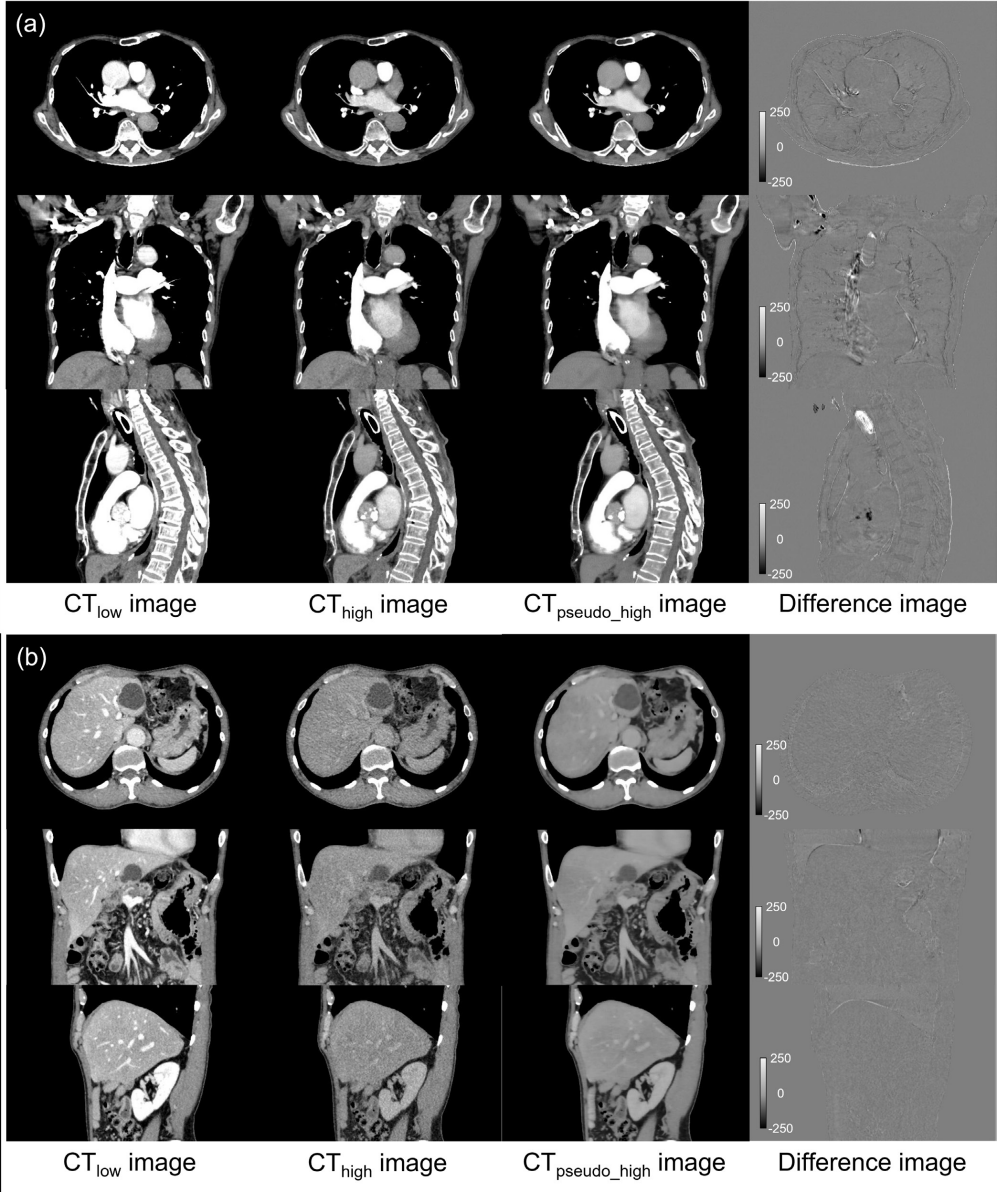
Examples of the CT_low_, CT_high_, and CT_pseudo_high_ images, and the differences between the CT_pseudo_high_ and CT_high_ images for patient 9 of the thoracic region and patient 11 of the abdominal region. The CT_low_, CT_high_, and CT_pseudo_high_ images are shown in a window of (50, 300) HUs. The images that show the differences are shown on a window of (−250, 250) HUs. (**a**) Thoracic region: the soft tissue in the CT_pseudo_high_ images are in good agreement with the CT_high_ images; however, some discrepancy can be seen in the high iodine concentration areas and bone edge. (**b**) Abdominal region: the CT_pseudo_high_ images agree well with the CT_high_ image. The image noise of CT_pseudo_high_ images is reduced in comparison with that of CT_high_ images.

Figure 2 a and b show examples of the CT_low_, CT_high_, and CT_pseudo_high_ images, and the differences between the CT_high_ and CT_pseudo_high_ images; the presented data correspond to patient 9 of the thoracic region group and to patient 11 of the abdominal region group. For the soft tissue of the thoracic region, CT_pseudo_high_ images, which are comparable with the CT_high_ images, can be generated; however, as observed in the coronal and sagittal views, some discrepancy has shown at high iodine concentration area and bone edge. In contrast, the CT_pseudo_high_ images are in good agreement with the corresponding CT_high_ images for the abdominal region. The image noise of CT_pseudo_high_ images improve in comparison with that of CT_high_ images. The image noise of CT_high_ and CT_pseudo_high_ images are 10.6 and 6.8 HU for the thoracic region; and 13.1 and 5.8 HU for the abdominal region, respectively. The differences in image noise are −3.9 and −7.3 HU for the thoracic and abdominal regions, respectively. The image noise is reduced significantly using the proposed model (*p* < 0.05).

The image noises of VNC_truth_ and VNC_pseudo_ images are 17.2 and 5.9 HU for the thoracic region, 12.5 and 5.7 HU for the abdominal region, respectively. The image noise is reduced significantly as well as CT images (*p* < 0.05)

### Quantitative evaluation metrics between ground truth and pseudo-images

Tables 1 and 2 show the comparison results of the CT value in the CT and VNC images. For the CT value of CT images, there are no significant differences between CT_high_ and CT_pseudo_high_ for all tissues. The mean differences in CT values are less than 6 HU. For the CT value of the VNC images, there are no significant differences between the VNC_truth_ and VNC_pseudo_ images, and the mean differences in the CT values are less than 10 HU.

**Table 1.** Comparison of CT values of CT and VNC images for thoracic region

Tissue	CT_high_ (HU)	CT_pseudo_high_ (HU)	Difference (HU)	*p*-value	VNC_truth_ (HU)	VNC_pseudo_ (HU)	Difference (HU)	*p*-value
Pulmonary artery	190.8 ± 48.4	188.0 ± 48.3	−2.7 ± 6.5	0.723	47.7 ± 11.7	43.6 ± 7.5	−4.1 ± 9.7	0.061
Pulmonary vein	156.8 ± 36.8	155.7 ± 33.9	−1.1 ± 9.9	0.678	43.5 ± 13.9	41.8 ± 5.1	−1.7 ± 11.7	0.539
Ascending aorta	142.5 ± 38.7	140.4 ± 37.3	−2.0 ± 6.8	0.051	40.6 ± 11.6	37.7 ± 4.2	2.9 ± 10.2	0.063
Lung	−861.5 ± 34.9	−860 ± 33.2	−1.3 ± 8.2	0.901	−872.2 ± 32.6	−870.1 ± 29.8	2.1 ± 11.8	0.566
Spine	290.5 ± 103.9	284.8 ± 104.6	−5.7 ± 13.0	0.783	213.6 ± 78.1	204.6 ± 77.8	−9.0 ± 19.8	0.406
Muscle	51.4 ± 7.6	49.2 ± 5.2	−5.2 ± 5.8	0.290	46.3 ± 8.4	46.7 ± 4.8	−5.5 ± 7.2	0.053

CT, Computed tomography; HU, Hounsfield unit; VNC, Virtual non-contrast.

**Table 2.** Comparison of CT values of CT and VNC images for abdominal region

Tissue	CT_high_ (HU)	CT_pseudo_high_ (HU)	Difference (HU)	*p*-value	VNC_truth_ (HU)	VNC_pseudo_ (HU)	Difference (HU)	*p*-value
Abdominal aorta	100.2.8 ± 9.6	100.0 ± 8.7	−0.3 ± 4.3	0.975	49.8 ± 7.2	49.2 ± 3.1	−0.6 ± 6.6	0.553
Portal vein	105.9 ± 13.1	106.9 ± 13.1	1.0 ± 8.0	0.723	50.0 ± 8.1	51.0 ± 9.2	1.0 ± 12.5	0.783
Liver	85.9 ± 13.4	85.4 ± 11.9	−0.5 ± 5.1	0.731	64.8 ± 11.2	64.2 ± 8.8	−0.6 ± 7.6	0.531
Spleen	87.6 ± 10.4	86.5 ± 7.9	−1.1 ± 5.9	0.706	56.2 ± 12.2	56.3 ± 4.9	1.0 ± 15.1	0.570
Pancreas	69.5 ± 11.5	69.6 ± 9.4	0.1 ± 5.2	0.889	47.9 ± 13.5	45.5 ± 7.0	1.0 ± 9.4	0.700
Kidney	109.9 ± 13.5	109.1 ± 14.8	−0.8 ± 5.2	0.639	48.1 ± 9.6	49.0 ± 6.0	−0.8 ± 8.2	0.315
Stomach	64.2 ± 12.6	65.7 ± 9.4	1.6 ± 7.4	0.665	46.0 ± 11.3	44.6 ± 6.8	2.9 ± 12.9	0.350

CT, Computed tomography; HU, Hounsfield unit; VNC, Virtual non-contrast.

Table 3 lists the MAEs and HIs in CT, VNC, and IM images for thoracic and abdominal regions. The mean MAEs for the CT and VNC images are less than 15 HU for all the patients that are being evaluated. The HIs are close to 1.000 for all the patients, and thus those results represent that the histogram of CT values of synthetic images are similar to those of ground truth images.

**Table 3.** Summary of evaluating metrics in the CT image, VNC image, and IM the thoracic and abdominal regions

Metrics	CT image		IM		VNC image	
	Thoracic region	Abdominal region	Thoracic region	Abdominal region	Thoracic region	Abdominal region
MAE (HU)	8.6 ± 1.7	6.2 ± 1.4	13.2 ± 5.0	6.8 ± 1.2	11.2 ± 2.1	8.1 ± 1.7
HI	0.9169 ± 0.0315	0.9543 ± 0.0250	0.8655 ± 0.050	0.8822 ± 0.025	0.9061 ± 0.0353	0.9246 ± 0.0293

CT, Computed tomography; HI, Histogram intersection; HU, Hounsfield unit; IM, Iodine map; MAE, Mean absolute error; VNC, Virtual non-contrast.

**Figure 3.**
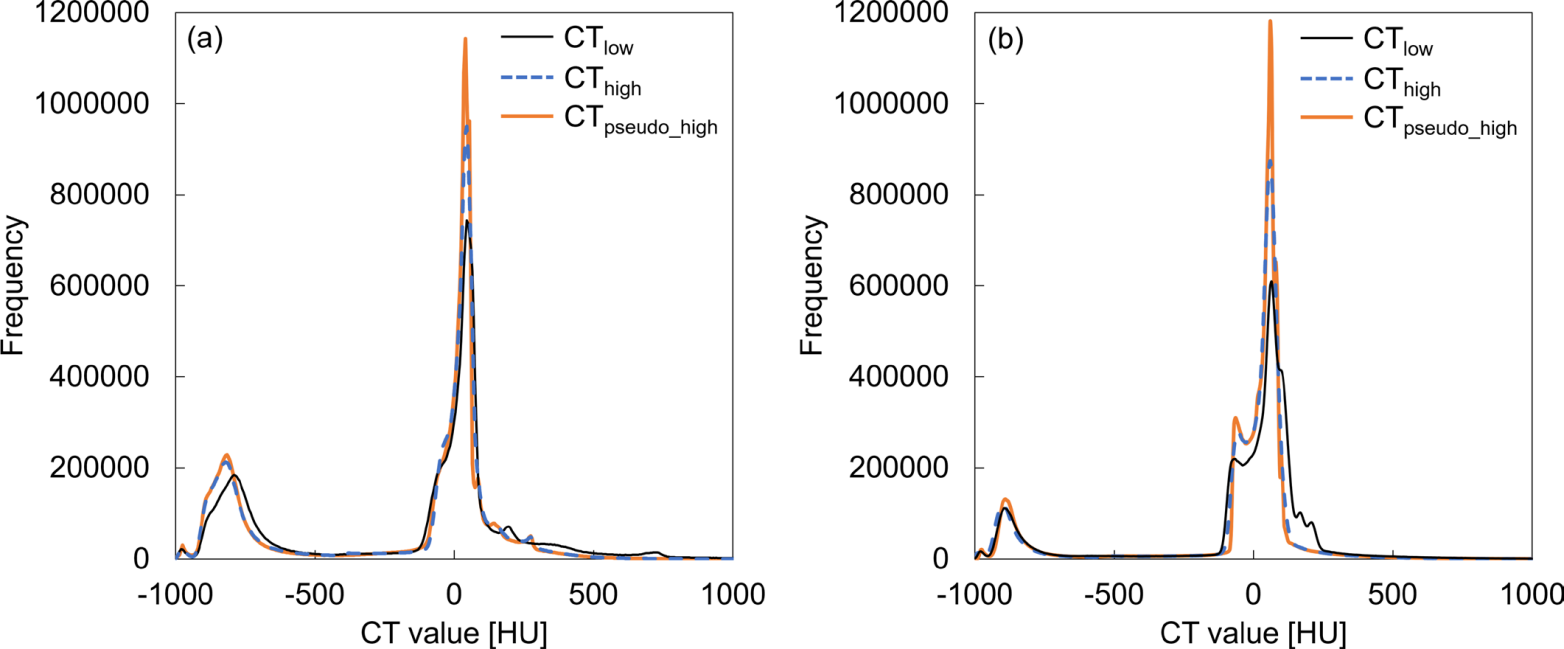
Examples of histograms of the CT_low_, CT_high_, and CT_pseudo_high_ images for (**a**) patient 9 of the thoracic group and (**b**) patient 11 of the abdominal group. As it can be seen in the histograms, there are some discrepancies at the peaks of around 0 HU; however, the histogram of the CT_pseudo_high_ images is in good agreement with that of the CT_high_ images for both regions.

Figure 3a and b show examples of image histograms for the 3D volume of the CT_low_, CT_high_, and CT_pseudo_high_ images; the presented data correspond to patient 9 of the thoracic group and to patient 11 of the abdominal group. There are some discrepancies at the peaks of around 0 HU; however, the histograms of the CT_pseudo_high_ images are in good agreement with that of CT_high_ images for both regions.

## Discussion

In this study, we developed a ResUnet-based method to yield CT_pseudo_high_, VNC_pseudo_ images, and IM_pseudo_ using only simple image processed CT_low_ images for thoracic and abdominal regions.

Some ResUnet-based methods have been reported, including those for predicting dose of radiotherapy and for CT image generation from magnetic resonance imaging (MRI).^10,21^ In the present study, we have successfully constructed the ResUnet model to generate the DECT imaging using only single energy CT image.

For the quantitative evaluation of the MAE values, previous studies have reported that the MAEs between the CT images generated using the MRI or the CBCT and ground truth CT images were 18.98–84.8 HU.^21,22^ Although the modalities were different, the MAEs in our study were lower than those in the previous studies. The accuracy of our proposed model was improved in comparison with previous reported model.

Several researchers have implemented CNN models in which high-quality DECT images were yielded from SECT images.^12,13^ However, previous studies did not perform the evaluation for the image similarity and quality of synthetic images. In this study, we evaluated the synthetic images using several image metrics and our model was comparable to another previous model. Although previous studies generated highly accurate CT_high_ images, they required multiple deep learning models and an indirect prediction approach using differential images. However, these complicated methods may not be suitable for clinical use. Our study introduces a novel approach that utilizes simple image-processing techniques, such as edge detection and thresholding, to perform data augmentation on the original images. Furthermore, the proposed methods yield direct predictions of CT_high_ images and achieve results comparable to those of previous studies.^12,13^ Therefore, the proposed approach has the potential to overcome the limitations of the the previous studies.

As observed in Figure 2, the CT_pseudo_high_ image noise reduced in comparison with that of the CT_high_ images. Schindera et al reported that an image noise reduction rate of 43.9–63.9% does not have a significant difference in the sensitivity for tumour detection.^23^ For our results of CT images, the noise reduction rate was approximately 25–55% for both thoracic and abdominal regions. It is possible that the proposed model can reduce the image noise without affecting the diagnosis. This reduction of image noise also affected the shape of histogram. The discrepancies of the histogram were caused by the reduction of image noise. However, the histograms show an almost identical shape between the ground-truth and synthetic images. Thus, the proposed model may prove to be helpful for noisy CT images.

**Figure 4.**
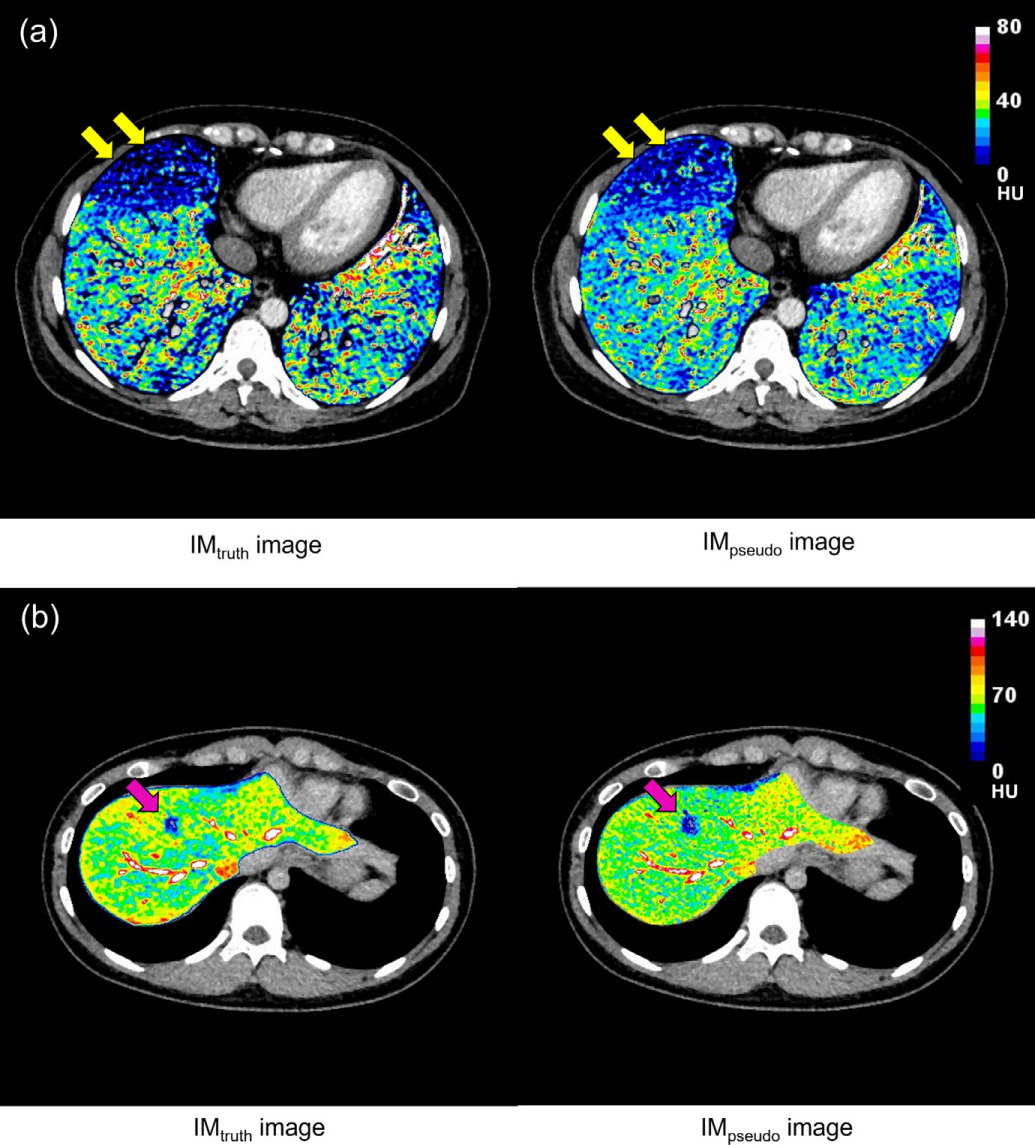
IM_truth_ and IM_pseudo_ for patient 5 of the thoracic group and patient 5 of the abdominal regions. (**a**) For the thoracic region, IM_pseudo_ can predict a change of perfusion as well as IM_truth_. (**b**) For the abdominal region, IM_pseudo_ provides the tumour location and iodine distribution as well as IM_truth_.

The present study also focused on obtaining VNC and IMs images. IMs has been reportedly used for surrogate images of lung perfusion and liver blood flow.^7,24^ The VNC image is generated by subtracting the IM from the contrast-enhanced CT image, and thus the VNC image is strongly related with IM.^25^ In the quantitative evaluation of the VNC image and IM, the MAEs in the present study were also found to be lower than those in previous study.^21,22^ For the VNC image and IM, the HIs are close to 1.000, which indicates that the similarity between the synthetic and ground truth images are high. Figure 4 shows the examples of IMs for thoracic and abdominal groups. According to a diagnostic report from an experienced radiologist, the patient in the thoracic region has pulmonary embolism (Figure 4a); the patient in the abdominal region is diagnosed with liver metastasis, which is caused by pancreatic cancer (Figure 4b). Although the IM_pseudo_ was not in perfect agreement with IM_truth_, the IM_pseudo_ could predict a perfusion change as well as IM_truth_. Therefore, our model showed the possibility of the generation of the IM_pseudo_ images similar to the IM_truth_ images.

The main advantage of the proposed model is that the CT_pseudo_high_ images can be acquired from only the image processed CT_low_ images. Using the proposed models, the DECT image can be generated using only a SECT image obtained by a single CT scan. Furthermore, DECT images can be obtained from single-phase CT scan data acquired in the past. Therefore, the proposed method may be valuable not only for institutions that have implemented only SECT but also for various other institutions with DECT. Hence, the proposed model could resolve several problems, including the increase in radiation dose, discrepancy in the contrast-enhancement phase, and adverse effect of the patient’s movement. The proposed model also enables DECT imaging without the installation of a special DECT scanner, which is advantageous for several institutions that may not have access to a special DECT scanner. Previous studies have reported that CT examinations using a low tube voltage contribute to a reduction in radiation dose and contrast agent volume.^26,27^ The proposed model can obtain the DECT image only using the CT_low_ image, thus, may contribute to reducing the required radiation dose and contrast agents.

The proposed ResUnet model has a simple architecture in comparison with other models, such as generative adversarial network.^28^ Once the trained ResUnet models are generated, the new CT_pseudo_high_ image can be yielded in a few minutes using the trained model. Therefore, DECT imaging with the developed model will be useful for clinical cases.

This study has two main limitations. First, the proposed models were trained and tested using only institutional CT data. Therefore, we used only the specified energy pair (thoracic and abdominal acquisition protocols at our institution), and multicentre studies with multiple energy pairs are needed in the future.

Second, the diagnostic ability of synthetic images and the impact of image quality (*e.g.,* image noise and artefacts) on the diagnosis could not be evaluated in this study. Therefore, in the future, further investigations are required on the effect of image quality on diagnostic performance.

## Conclusions

In this study, we successfully developed a model to yield DECT_pseudo_ image from only simple image processed CT_low_ images. Our comparison results indicated that the proposed model enabled to generate CT_pseudo_high_ similar to CT_high_ images. Additionally, it was found that the image noise was reduced using the proposed model. The VNC_pseudo_ images and IM_pseudo_ could be generated in a manner similar to IM_truth_ and VNC_truth_ using the CT_pseudo_high_ and CT_low_ images. The results suggest that the proposed model enabled to obtain the DECT images and to provide the material-specific images from only SECT images.
