# Supplementary material for: Pseudo dual-energy CT-derived iodine mapping using single-energy CT data based on a convolution neural network
Source: BJR Open. 2023 Oct 18;5(1):20220059. doi: 10.1259/bjro.20220059 (PMC10630979; doi:10.1259/bjro.20220059)
Supplement: Supplementary file 1 — Supplementary S1. [file bjro.20220059.suppl-01.docx]

**Supplementary Material 1**

**Implementation detail of proposed ResUnet model**

Figure S1 (a) shows the architecture of the ResUnet model used in the proposed method. This model was implemented in Keras (ver.2.2.4) with a TensorFlow backend. For the model architecture, the three-channel images were input and a one-channel image was yielded as the output. The proposed model consisted of 13 residual blocks. Figure S1 (b) shows the construction of the residual blocks for up-sampling and down-sampling of the feature map. The spatial resolutions of the feature map of the input data were reduced from 512 × 512 to 4 × 4 pixels using 7 down-sampling residual blocks. Next, those of the feature map were increased from 4 × 4 to 512 × 512 pixels using 6 up-sampling residual blocks. The down-sampling residual block consisted of two two-dimensional (2D) convolution layers with a kernel size of 3 × 3 pixels and stride of 1 pixel, two batch-normalization layers, a max pooling layer with a window size of 2 × 2 pixels, and two rectified liner unit (ReLU) activation layers.

The up-sampling residual block consisted of a 2D deconvolution layer with a kernel size of 2 × 2 pixels and strides of 2 pixels, two 2D convolution layers with a kernel size of 3 × 3 pixels and stride of 1 pixel, two batch-normalization layers, two ReLU activation layers, and a dropout layer. To prevent the model from over-fitting, a dropout layer with a dropout rate of 0.5 was added before the output. The shortcut connection was used for both residual blocks.

For the model implementation, the mean absolute error (MAE) was used as the loss function:

$MAE=\frac{1}{N}\sum_{i=1}^{N} \left| {CT}_{pseudo\_high}(i)-{CT}_{high}(i) \right|$ (1)


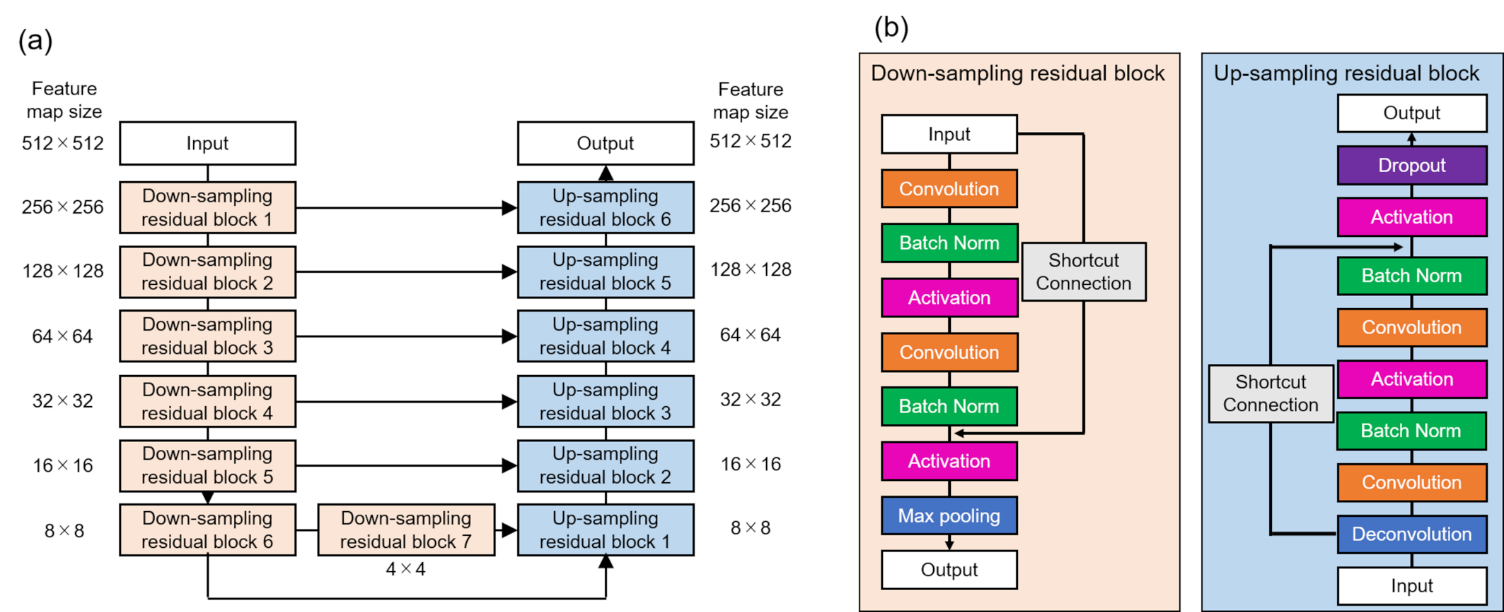
where *N* is the number of pixels, *CT_pseudo_high_* is the pixel value of the CT_pseudo_high_ image, and *CT_high_* denotes the pixel value of the CT_high_ image. The loss function was minimized using the Adam algorithm ^25^; the initial learning rate was 0.001. The mini-batch size and the epoch size were 12 and 50, respectively. Training was performed using the early stopping module of Keras to avoid model overfitting.

Figure S1 (a) Architecture of the ResUnet model. The ResUnet model consists of 13 residual blocks. (b) The detail of Residual blocks used for down-sampling and up-sampling.

**Training results of proposed ResUnet model**

Figure S2 shows the results of the training and validation of the thoracic and abdominal regions. The loss functions of the training data decreased with increasing epochs. Similarly, those of the validation data also decreased with increasing epochs for both the thoracic and abdominal regions. These results suggest that the proposed model does not overfit the training data.


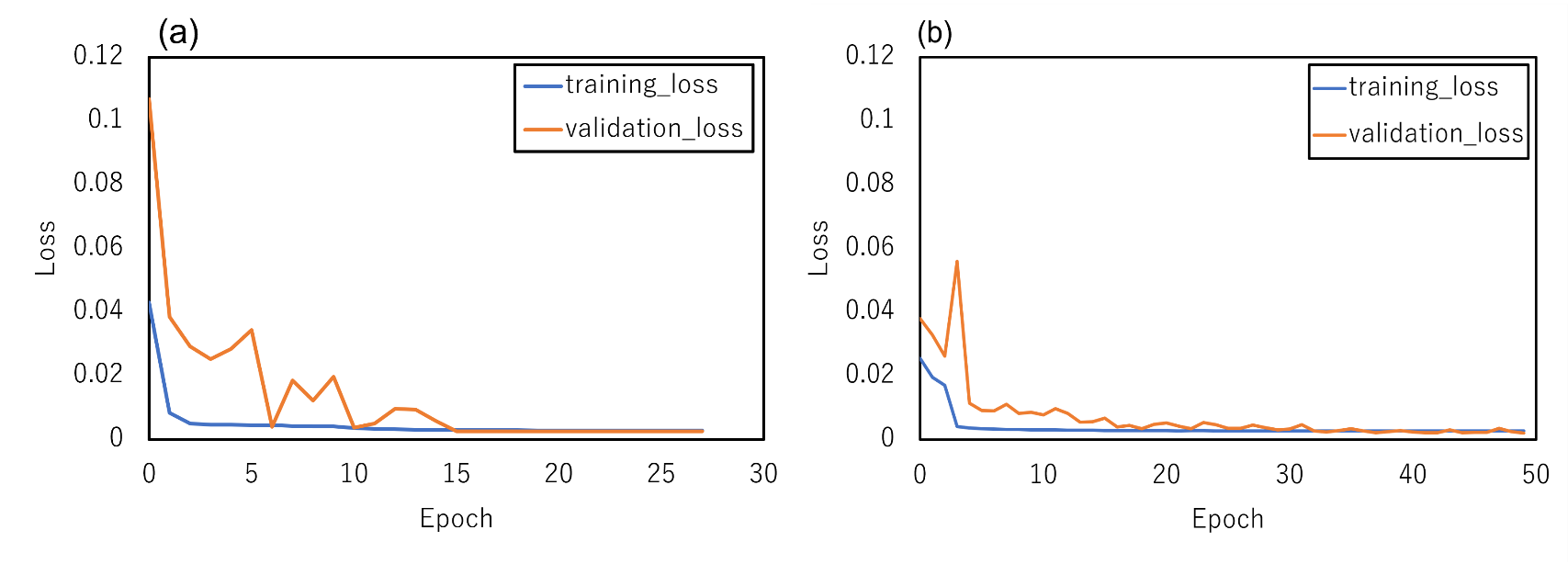
Figure S2 The training and validation results for (a) thoracic and (b) abdominal regions. The loss functions of the training data decreased with increasing epochs for both the thoracic and abdominal regions. The values of the validation data also decreased with increasing epochs. The early stopping module of Keras was used to avoid overfitting.
